# Metabolic Risk Factors of Type 2 Diabetes Mellitus and Correlated Glycemic Control/Complications: A Cross-Sectional Study between Rural and Urban Uygur Residents in Xinjiang Uygur Autonomous Region

**DOI:** 10.1371/journal.pone.0162611

**Published:** 2016-09-13

**Authors:** Guo-li Du, Yin-xia Su, Hua Yao, Jun Zhu, Qi Ma, Ablikm Tuerdi, Xiao-dong He, Li Wang, Zhi-qiang Wang, Shan Xiao, Shu-xia Wang, Li-ping Su

**Affiliations:** 1 Department of Endocrinology, the First Affiliated Hospital of Xinjiang Medical University, Urumqi, China; 2 The Key Laboratory of Xinjiang Metabolic Disease, Clinical Medical Research Institute, the First Affiliated Hospital of Xinjiang Medical University, Urumqi, China; 3 Department of Occupational and Environmental Health, Xinjiang Medical University, Urumqi, China; 4 Department of Health Check Centre, the First Affiliated Hospital of Xinjiang Medical University, Urumqi, China; 5 Department of Pathology, the First Affiliated Hospital of Xinjiang Medical University, Urumqi, China; Shanghai Diabetes Institute, CHINA

## Abstract

**Background:**

Diabetes is a major global public health problem driven by a high prevalence of metabolic risk factors.

**Objective:**

To describe the differences of metabolic risk factors of type 2 diabetes, as well as glycemic control and complicated diabetic complications between rural and urban Uygur residents in Xinjiang Uygur Autonomous Region of China.

**Methods:**

This comparative cross-sectional study, conducted among 2879 urban and 918 rural participants in Xinjiang, China, assessed the metabolic risk factors of diabetes and related complications differences between urban and rural settlements.

**Results:**

Compared to rural areas, urban participants had higher education level and more average income, little physical activity, less triglycerides and higher HDL-c (p *<* 0.05 respectively). Differences in metabolic risk factors by urban/rural residence included overweight or obesity, triglycerides (≥1.71mmol/l), HDL-c (< 1.04 mmol/l), alcohol intake, and physical inactivity (p *<* 0.01 respectively). There was significant difference regarding the prevalence of HbA1c >8% (48.1% versus 54.5%, p *=* 0.019) between rural and urban diabetic participants. No significant difference in the prevalence of type 2 diabetic complications between urban and rural participants (74.9% versus 72.2%; p *=* 0.263) was detected. Compared to rural participants, the most prevalent modifiable risk factors associated with diabetic complications in urban participants were obesity (BMI ≥ 28 Kg/m^2^), HDL-c (< 1.04 mmol/l), physical inactivity and irregular eating habits (p *=* 0.035, p = 0.001, p < 0.001, and p = 0.013, respectively).

**Conclusions:**

Urban settlers were significantly more likely to have metabolic risk factors highlighting the need for public health efforts to improve health outcomes for these vulnerable populations. Diabetes related complications risk factors were prevalent amongst rural and urban diabetes settlers.

## Introduction

Type 2 diabetes mellitus (T2DM), as the fourth major cause of mortality worldwide, is a major public health problem presenting a significant rising prevalence [1,2]. It is a complex metabolic disease mostly resulting from the interaction among genetic, environmental and other risk factors. Risk factors for T2DM include obesity [3], sedentary lifestyle [4], smoking [5], high fat/cholesterol diet [6] and refined carbohydrates [7], and some psychological factors [1,3]. This metabolic disease requires effective long-term management to achieve optimal glycemic control and minimize chronic complications [8–10].

In developing countries, with rapid western cultural adaptation and urbanization [9,11], diabetes prevalence is rising dramatically [12], accompanied with the rising burden of this condition [13–16]. In China, it was reported that the prevalence of T2DM among people older than 20-year-old was 9.7% and more common in the capital city [17–19]. For example, an isolated study reported that the prevalence of T2DM had been increasing sharply and the prevalence of diabetes increased from 9.7% to 12.6% between 2002 and 2009 in Shanghai, the largest city of China with rapid urbanization [20].

Previous study showed that the prevalence of diabetes was higher in urban population than rural population [4,6,21], and a higher prevalence [22] increased in rural communities [12,23]. Diabetes affects 14.1% people living in urban areas in China [20]. Urban populations often live with better socioeconomic conditions, higher level of education and less physical activity compared with rural populations [24].

Bordering eight countries including Russia, Kazakhstan, Kyrgyzstan, Tajikistan, Pakistan, Mongolia, India, and Afghanistan, Xinjiang Uygur Autonomous Region is located in the northwest of China and composed of more than 13 ethnic groups, with Uygur accounting for 46%, Han accounting for 40%, and Kazak accounting for 7%. Originating from inter-marriage between Caucasians and Mongolians, the Uygur people have their own language, culture, genetic background, lifestyle, and dietary habits. Previous reports showed differences between Uygur and Han regarding the prevalence of T2DM [25] and genetic polymorphism [26,27]. The current study sought to assess the differences in prevalence of T2DM and metabolic risk factors among general and diabetic populations and their variations across compared populations in Xinjiang, China, drawn from urban and rural Uygur settlements.

## Materials and Methods

### Study population

This comparative cross-sectional study was conducted among 2897 urban and 918 rural participants ≥ 20 years of age, both located in Xinjiang Uygur Autonomous Region (Kashgar and Urumqi), from March 2012 to December 2013.

Participants were enrolled in the current study via simple random sampling technique. A representative sample of the general populations of Uygur were chosen and classified as urban and rural groups based on the government record of registered residence, settlement, location and way of life of the people [28].

Pregnant women, individuals with type 1 diabetes, MODY, stress hyperglycemia, malignant tumor, autoimmune disease, physically or mentally disabled persons and those with incomplete data were excluded.

### Anthropometric measurements

An interview-based questionnaire was used to gather information on socio-demographic and metabolic risk factors. It was designed in the Chinese language but translated into Uygur language for those who didn’t understand Chinese. The questionnaire collected information including demographic and socioeconomic status and medical history.

Family monthly income was used to evaluate household socioeconomic status. Food consumption over the past 7 days was calculated to analyze the eating habits.

Body weight, height, waist circumference and blood pressure measurements were performed using standardized methods. Body weight was measured to the nearest 0.1 Kg in light clothing with a mechanical scale. Height was measured to the nearest 0.1 cm without shoes, with a commercial stadiometer (HW-900B OMRON, Japan).

With the subjects standing and breathing normally, using a measuring tape parallel to the floor, waist circumference (WC) was measured at midpoint between the last palpable rib and the suprailiac crest, and the hip circumference (HC) was measured at the outermost points of the greater trochanters [6,29]. The formula; weight (Kg) / height (m^2^) was used to calculate the body mass index (BMI), WC (cm) / HC (cm) was used to calculate waist-to-hip ratio (WHR), and WC (cm) / height (cm) was used to calculate the waist-to-height ratio (WHtR).

Blood pressure was measured three times with a 5 minutes interval, by tail cuff method on the upper left arm with the patient in sitting position and after at least 5 minutes of rest [30]. The mean value of the last two measurement was used for analysis. Hypertension was defined as systolic pressure (SBP) ≥ 140 mmHg and/or diastolic pressure (DBP) ≥ 90 mmHg or self-reported use of antihypertensive medications irrespective of measured blood pressure.

### Occupation

Occupational data gathered were classified as formal, informal and unemployed based on set criteria. Those jobs with normal hours, regular wages recognized as income sources on which income taxes must be paid were considered as “formal” jobs. “Informal” jobs were defined as any such contrary to this.

### Biochemical assays

After observing an overnight fast (12–14 hours), blood specimen was collected for biochemical measurements. For each subject with no history of T2DM, an oral glucose tolerance test was taken, using a glucose load containing the equivalent of 75 g anhydrous glucose dissolved in water. T2DM was diagnosed according to measured glucose levels by WHO 1999 criteria [31], with a fasting plasma glucose (FPG) level ≥ 7.0 mmol/l after a minimum overnight 12 h fast, or a 2 h post-glucose level ≥ 11.1 mmol/l during an OGTT with symptoms of diabetes. In the absence of classic symptoms of hyperglycemia, results should be confirmed by repeating test in another day.

Blood glucose, as well as, glycosylated hemoglobin (HbA1c), total cholesterol (TC), triglyceride (TG), high-density lipoprotein cholesterol (HDL-c) and low-density lipoprotein cholesterol (LDL-c) were measured using the Dimension AR/AVL Clinical Chemistry System (Newark, NJ, USA) in the central laboratory of the First Affiliated Hospital of Xinjiang Medical University. Hyperlipidemia was defined based on lipids levels with TG ≥ 1.71mmol/l or/and LDL-c ≥ 3.38 mmol/l, or/and TC ≥ 5.20 mmol/l.

### Determination of T2DM related complications

CHD was identified from the linked data set using all diagnosis fields, and the presence of CHD was determined by an expert cardiologist [32,33]. Cerebrovascular disease was evaluated and determined by the specialist in Neurological department [19]. Screening for peripheral arterial disease (PAD) including a history for claudication and an assessment of the pedal pulses (the ankle-brachial index, ABI) was performed before the diagnosis [34,35]. Retinal vascular changes were evaluated with a dilated fundus examination followed by fundus fluorescein angiography (FFA), and those allergic to contrast agent would be evaluated by fundus photography. The diagnosis of diabetic peripheral neuropathy was based on the medical history and simple clinical tests including 10-g monofilament testing and at least one of the following tests: pinprick, temperature, or vibration sensation. Electrophysiological testing or referral to a neurologist was advised in situations where the clinical features were atypical or the diagnosis was unclear [1]. The diagnosis of diabetic nephropathy was based on urine albumin excretion, serum creatinine and estimated GFR [36,37].

### Statistical methods

Statistical was analyzed using SPSS16.0. Most of the variables were analyzed through descriptive statistics (Median (IQR) and percentage). Categorical variables were compared using the Chi-square or Fisher’s test. Multivariate logistic regression model was built to analyze the association of metabolic risk factors with urban residence, adjusted for age and gender. The associations between various variables within each population group was determined using Spearman’s rho (Rank) correlation analysis. It was considered statistically significant as p *<* 0.05.

### Ethical consideration

The study was conducted in accordance with the Declaration of Helsinki guidelines and approved by the Ethics Committee of the First Affiliated Hospital of Xinjiang Medical University. Participation was voluntary and written informed consent was obtained from the participants.

## Results

### Population characteristics

The socio-demographic characteristics of the study populations are shown in Table 1. There were significant differences between urban and rural participants with respect to gender distribution (p < 0.001) and mean age (p < 0.001). The participants in the urban area lived with higher average income, compared to that observed amongst participants in the rural area (p < 0.001). There were statistically significant differences in the marital status (p *=* 0.004), employment (p < 0.001), education (p < 0.001), physical activity on regular basis (p < 0.001) and alcohol intake (p < 0.001). No significant differences regarding intensity of performed physical activity, eating habits, reached recommended amount of fruits and vegetables/day and smoking were found between urban and rural participants (p *=* 0.840, 0.309, 0.411 and 0.961 respectively).

**Table 1:** Socio-demographic characteristics of the population.

Variables	Total (n = 3797)	Rural (n = 918)	Urban (n = 2879)	p-value
**Age (years)**				
**Median (IQR)**	46.0 (38.0–55.0)	49.0 (40.0–58.0)	44.7 (37.3–53.2)	< 0.001
20–29 yrs n (%)	317 (8.3)	69 (7.5)	248 (8.6)	
30–39 yrs n (%)	867 (22.8)	152 (16.6)	715 (24.8)	
40–49 yrs n (%)	1182 (31.1)	248 (27.0)	934 (32.4)	
50–59 yrs n (%)	847 (22.3)	253 (27.6)	594 (20.6)	
60+ yrs n (%)	584 (15.4)	196 (21.4)	388 (13.5)	< 0.001
**Gender n (%)**				
Male	2166 (57.0)	573 (62.4)	1593 (55.3)	
Female	1631 (43.0)	345 (37.6)	1268 (44.7)	< 0.001
**Family history of diabetes n (%)**				
No	2942 (77.5)	733 (79.8)	2209 (76.7)	
Yes	855 (22.5)	185 (20.2)	670 (23.3)	0.049
**Marital status n (%)**				
Single	238 (6.3)	40 (4.4)	198 (6.9)	
Married	3487 (91.9)	852 (92.8)	2635 (91.6)	
Divorced	31 (0.8)	12 (1.3)	19 (0.7)	
Widowed	40 (1.1)	14 (1.5)	26 (0.9)	0.004
**Occupation n (%)**				
Formal	2585 (68.2)	488 (49.0)	2137 (74.3)	
Informal	466 (12.3)	228 (24.9)	238 (8.3)	
Unemployed	740 (19.5)	238 (26.0)	502 (17.4)	< 0.001
**Level of education n (%)**				
Illiterate	78 (2.1)	51 (5.6)	27 (0.9)	
Basic	294 (7.8)	156 (17.1)	138 (4.8)	
Secondary education	1411 (37.2)	440 (48.1)	971 (33.8)	
Tertiary	2007 (53.0)	267 (29.2)	1740 (60.5)	< 0.001
**Average income (RMB) n (%)**				
<2000	733 (19.3)	321 (35.1)	412 (14.3)	
2000~4000	1398 (63.2)	519 (56.8)	1879 (65.3)	
4000~6000	562 (14.8)	60 (6.6)	502 (17.4)	
6000~8000	72 (1.9)	11 (1.2)	61 (2.1)	
≥8000	27 (0.7)	3 (0.3)	24 (0.8)	< 0.001
**Smoking n (%)**				
No	2598 (68.9)	631 (68.8)	1967 (68.9)	
Yes	1174 (31.1)	286 (31.2)	888 (31.1)	0.961
**Alcohol intake n (%)**				
No	2877 (76.8)	740 (81.6)	2137 (75.3)	
Yes	868 (23.2)	167 (18.4)	701 (24.7)	< 0.001
**Physical activity on regular basis**				
No	2273 (59.9)	310 (33.8)	1214 (42.2)	
Yes	1524 (40.1)	608 (66.2)	1665 (57.8)	< 0.001
**Intensity of performed physical activity**				
High	114 (7.5)	21 (6.8)	93 (7.7)	
Moderate	77 (5.1)	15 (4.8)	62 (5.1)	
Low	1330 (84.7)	274 (88.4)	1056 (87.2)	0.840
**Eating habits**				
3 main courses/day	3143 (82.9)	771 (84.0)	2372 (82.5)	
Irregular habits	649 (17.1)	147 (16.0)	502 (17.5)	0.309
**Reached recommended amount of fruits and vegetables/day**				
No	705 (19.1)	182 (20.0)	523 (18.8)	
Yes	2992 (80.9)	728 (80.0)	2264 (81.2)	0.411

Data is presented as median (IQR) or n(%); Chi-square or Fisher’s test. p < 0.05 was considered significant difference. n: number. Line45: Only participants reporting regular physical activity included in this analysis, ≥ 5 times/week at moderate or high intensity, Line 55: 500 grams of fruits and vegetables/day.

### Metabolic risk factors in urban and rural population

Among participants, systolic and diastolic BP median (IQR) was 121(111–130) and 83(70–90) mmHg, respectively, and rural participants had higher blood pressure compared to urban participants. As shown in Table 2, regarding hip circumference and WHR, participants in the urban area had significantly higher measures (p < 0.001 respectively). There were no significant differences in BMI between urban and rural people (p = 0.229). Participants in the rural area had significantly higher levels of serum triglyceride levels than those participants in the urban area (p < 0.001). For HDL-c, the urban participants were higher than rural participants (p < 0.001) [Table 2].

**Table 2:** Anthropometric variables and serum lipid levels of the study population, median (IQR) or n (%).

Variables	Total (n = 3797)	Rural (n = 918)	Urban (n = 2879)	p-value
**FBS**	5.39 (4.72–7.81)	6.61 (5.01–9.25)	5.21 (4.67–7.33)	< 0.001
**Type 2 Diabetes**	1507(39.7)	513(55.9)	994(34.5)	< 0.001
**Pre-diabetes**	924(24.3)	340(37.0)	584(20.3)	< 0.001
**Hypertention**	1317(34.7)	397(43.4)	920(32.0)	< 0.001
**SBP (mmHg)**	122 (111–130)	124 (115–130)	121 (110–130)	< 0.001
**DBP (mmHg)**	83 (70–90)	85 (72–92.5)	82 (70–90)	< 0.001
**Weight (Kg)**	76(67–84)	75(66–84)	76.5 (68–83.5)	0.239
**Height (cm)**	168(163–172)	168(162–173)	168 (163–172)	0.922
**BMI (Kg/m**^**2**^**)**	26.47 (24.27–29.05)	26.44 (23.99–29.29)	26.53 (24.39–29.03)	0.229
**Obesity (BMI ≥ 28 Kg/m**^**2**^**)**	1283(33.9)	311(33.9)	972(33.8)	0.985
**WC**	95.0 (88.5–102)	96 (88–104)	95 (88.5–102.0)	0.067
**HC**	100.0 (85.0–108.0)	102 (93–110)	103.00 (95–109.00)	< 0.001
**WHR**	0.94 (0.89–1.07)	0.93 (0.89–1.01)	0.95 (0.88–1.11)	< 0.001
**Hperlipidemia**	2957(77.0)	780(85.0)	2177(75.6)	< 0.001
**TC (mmol/l)**	4.31 (2.95–5.12)	4.2 (2.76–5.05)	4.34 (3.04–5.15)	0.078
**TG (mmol/l)**	1.93 (1.17–4.03)	2.05 (1.27–4.13)	1.85 (1.13–3.94)	< 0.001
**HDL-c (mmol/l)**	1.12 (0.92–1.34)	1.03 (0.83–1.26)	1.15 (0.95–1.36	< 0.001
**LDL-c (mmol/l)**	2.77 (2.31–3.32)	2.78 (2.26–3.32)	2.77 (2.33–3.32)	0.705

Data is presented as median (IQR) or n (%); Chi-square or Fisher’s test. p < 0.05 was considered significant difference. IQR: Interquartile range, BMI: Body Mass Index, WC: Waist Circumference, HC: Hip Circumference, WHR: Waist to Hip Ratio, TC: Total Cholesterol, TG: Triglycerides, HDL-c: High Density Lipoprotein cholesterol, LDL-c: Low Density Lipoprotein cholesterol.

In Table 3, metabolic risk factors between rural and urban participants were compared. The prevalence of hypertension was 43.4% in rural residents, higher than that in urban residents (32.0%, p < 0.001). The prevalence of overweight (BMI ≥ 25 Kg/m^2^) and obesity (BMI ≥ 28 Kg/m^2^) were greater in urban participants than that in rural participants (p < 0.001 and p < 0.001 respectively). For hyperlipidaemia, the prevalence of higher triglyceride (≥ 1.71 mmol/l) and less HDL cholesterol (< 1.04 mmol/l) were observed higher among rural participants (p = 0.002 and p < 0.001 respectively). There were no differences between rural and urban residents regarding TC ≥ 5.20 mmol/l (21.8% versus 23.6%, p = 0.265) and LDL-c ≥ 3.38 mmol/l (23.1% versus 23.3%, p = 0.920). In contrast, participants in the urban area were more likely than rural residents, to have more alcohol intake, as well as less physical inactivity (p < 0.001 and p = 0.002 respectively).

**Table 3:** Metabolic risk factors in urban population compared with rural population.

Risk factors	Crude OR (95% CI)	Total (n = 3797) n (%)	Rural (n = 918) n (%)	Urban (n = 2879) n (%)	p-value
**Hypertension**	0.613 (0.527–0.715)	1317 (34.8)	397 (43.4)	920 (32.0)	<0.001
**BMI(≥ 25 Kg/m**^**2**^**)**	2.637 (2.097–3.317)	2128 (84.9)	450 (74.1)	1678 (88.3)	<0.001
**BMI (≥ 28 Kg/m**^**2**^**)**	2.210 (1.737–2.813)	1283 (77.2)	311 (66.5)	972 (81.4)	<0.001
**WC (men ≥ 90 cm; women ≥ 85 cm)**	1.039 (0.868–1.243)	2972 (78.4)	715 (77.9)	2257 (78.5)	0.679
**WHR (men > 0.90; women > 0.85)**	0.813 (0.676–0.977)	2944 (77.6)	737 (80.3)	2207 (76.8)	0.027
**TC (≥ 5.20 mmol/l)**	1.107 (0.926–1.324)	879 (23.2)	200 (21.8)	679 (23.6)	0.265
**LDL-c (≥3.38 mmol/l)**	1.009 (0.846–1.204)	882 (23.2)	212 (23.1)	670 (23.3)	0.920
**HDL-c (< 1.04 mmol/l)**	0.517 (0.445–0.601)	1472 (38.8)	467 (31.7)	1005 (34.9)	<0.001
**TG (≥ 1.71mmol/l)**	0.785 (0.675–0.913)	2097 (55.3)	548 (59.8)	1549 (53.8)	0.002
**Alcohol intake**	1.454 (1.204–1.755)	868 (23.2)	167 (18.4)	701 (24.7)	<0.001
**Smoking**	0.996 (0.848–1.170)	1174 (31.1)	286 (31.2)	888 (31.1)	0.961
**Physical inactivity**	0.699 (0.599–0.817)	2273 (59.9)	608 (66.2)	1665 (57.8)	<0.001
**Irregular eating habits**	0.110 (0.908–1.357)	649 (17.1)	147 (16.0)	502 (17.5)	0.309

Data is presented as n (%); OR: Odds ratio. Compared using multivariate logistic regression. p < 0.05 was considered significant difference. BMI: Body Mass Index, WC: Waist Circumference, WHR: Waist to Hip Ratio, TC: Total Cholesterol, LDL-c: Low Density Lipoprotein cholesterol, HDL-c: High Density Lipoprotein cholesterol, TG: Triglycerides.

In Table 4, SBP correlated positively with DBP and so was waist circumference with WHR, WHtR, total cholesterol, LDL-c and HDL-c in participants of both urban and rural areas. A significant positive correlation between DBP and BMI was observed only in the urban participants. There were also significant positive correlations between BMI and WC, WHR, total cholesterol, triglycerides, LDL-c and HDL-c both in rural and urban participants and so was total cholesterol with triglycerides and LDL-c. LDL-c showed a significant positive association with FBS and HbA1c in urban participants but not in rural participants. FBS showed a significant positive association with HC amongst participants only in urban participants.

**Table 4:** Spearman’s rho correlation coefficients between selected metabolic variables for rural (Lower Left-Hand Side) and urban (Upper Right-Hand Side).

**Variables**	**SBP**	**DBP**	**FBS**	**HbA1c**	**BMI**	**WC**	**HC**	**WHR**	**WHtR**	**TC**
**SBP**	1	0.416**	0.206**	-0.058	0.189 **	0.123**	0.212 **	-0.050**	0.132**	0.096**
**DBP**	0.306 **	1	0.012	-0.103**	0.075**	-0.049**	0.293**	-0.278**	-0.040*	-0.345**
**FBS**	0.221 **	-0.204**	1	0.506 **	0.178**	0.239 **	0.250 **	0.049**	0.216**	0.173**
**HbA1c**	-0.037	-0.153**	0.508**	1	-0.107 **H7	-0.032	-0.013	0.001	-0.007	0.100*
**BMI**	0.141 **	0.011	0.086*	0.014	1	0.558 **	0.202**	0.234**	0.580**	0.108**
**WC**	0.092 **	-0.075*	0.141**	-0.026	0.607 **	1	0.289 **	0.445**	0.926**	0.179**
**HC**	0.068 *	0.083*	0.059	0.013	0.436 **	0.624 **	1	-0.629**	0.280**	-0.131**
**WHR**	0.087 **	-0.181**	0.185**	-0.051	0.200 **	0.408 **	-0.318**	1	0.404**	0.290**
**WHtR**	0.104**	-0.085**	0.140**	-0.002	0.619 **	0.937 **	0.573 **	0.397**	1	0.174**
**TC**	0.224 **	-0.283**	0.315**	0.082	0.112 **	0.145 **	-0.042	0.262**	0.152**	1
**TG**	-0.066*	0.393 **	-0.168**	0.032	0.114 **	0.055	0.155**	-0.103**	0.048	-0.220**
**LDL-c**	0.083*	0.151 **	-0.013	0.06	0.089**	0.088 **	0.072*	0.043	0.088**	0.495**
**HDL-c**	-0.133**	0.182 **	-0.482**	0.021	-0.150 **	-0.175 **	-0.090**	-0.186**	-0.154**	-0.061
**EH**	0.024	-0.048	0.115 **	0.133*	0.025	0.042	0.012	0.091**	0.03	0.077*
**EDU**	-0.134**	0.003	-0.345**	-0.143*	0.046	0.016	-0.056	0.011	-0.052	-0.107**
**OCP**	0.217**	-0.162**	0.431 **	0.109	-0.014	-0.003	-0.008	0.081*	0.049	0.232**
**AI**	0.078*	-0.059	-0.055	-0.180**	0.123**	0.126**	0.058	0.093**	0.081*	0.013
**Smoking**	-0.016	0.079*	-0.166**	-0.124*	0.076*	0.129**	0.097**	0.018	0.026	-0.119**
**AlI**	0.001	0.066*	-0.125**	-0.098	0.104**	0.134**	0.119**	-0.015	0.066*	-0.073*
**PIA**	0.045	-0.092**	0.120**	-0.124*	-0.031	-0.089**	-0.148**	0.070*	-0.070*	0.148**
**Variables (continued)**	**TG**	**LDL-c**	**HDL-c**	**EH**	**EDU**	**OCP**	**AI**	**Smoking**	**AlI**	**PIA**
**SBP**	0.021	0.058**	-0.117**	0.056**	-0.124**	0.173**	-0.005	0.008	0.042*	-0.027
**DBP**	0.446**	0.051**	0.017	-0.045*	-0.214**	-0.005	-0.070**	0.035	-0.005	-0.009
**FBS**	0.068**	0.055**	-0.400**	0.108**	-0.264**	0.305**	0.070**	0.025	0.029	0.041*
**HbA1c**	0.073	0.082*	-0.008	0.158**	-0.154**	0.065	-0.071	-0.033	-0.001	-0.103**
**BMI**	0.081**	0.117**	-0.186**	0.018	-0.042*	0.061**	0.062**	0.066**	0.075**	0.004
**WC**	0.009	0.112**	-0.250**	0.048**	-0.036	0.091**	0.088**	0.182**	0.176**	0.003
**HC**	0.270**	0.139**	-0.140**	0.053**	-0.204**	0.125**	-0.027	0.062**	0.031	0.009
**WHR**	-0.248**	-0.033	-0.114**	0.029	0.116**	-0.011	0.102**	0.102**	0.122**	-0.009
**WHtR**	0	0.117**	-0.185**	0.033	-0.065**	0.131**	0.051**	0.071**	0.076**	0.009
**TC**	-0.237**	0.530**	0.007	0.092**	0.164**	0.024	0.116**	0.041*	0.103**	0
**TG**	1	0.219**	-0.169**	-0.053**	-0.176**	-0.068**	-0.045*	0.167**	0.086**	-0.011
**LDL-c**	0.249**	1	0.060**	0.016	-0.002	-0.009	0.027	0.054**	0.076**	0.004
**HDL-c**	-0.03	0.196**	1	-0.079**	0.177**	-0.157**	-0.069**	-0.191**	-0.162**	-0.002
**EH**	-0.032	-0.015	-0.102**	1	-0.022	0.008	0.096**	0.049**	0.063**	0.071**
**EDU**	0.063	-0.012	0.182**	-0.094**	1	-0.341**	0.211**	0.027	0.077**	-0.052**
**OCP**	-0.205**	-0.019	-0.186**	0.016	-0.501**	1	-0.171**	-0.128**	-0.120**	0.032
**AI**	-0.029	0.002	-i0.074*	-0.036	0.336**	-0.241**	1	0.078**	0.129**	0.042*
**Smoking**	0.182**	0.04	-0.049	0.007	0.233**	-0.261**	0.134**	1	0.535**	-0.011
**AlI**	0.157**	0.029	-0.065	-0.004	0.195**	-0.236**	0.163**	0.506**	1	-0.062**
**PIA**	-0.139**	-0.027	-0.048	-0.034	-0.047	0.06	0.06	-0.088**	-0.105**	1

** Correlation is significant at the 0.01 level (2-tailed).

* Correlation is significant at the 0.05 level (2-tailed).

Abbreviations: SBP: Systolic Blood Pressure, DBP: Diastolic Blood Pressure, FBS: Fasting Blood Sugar, WC: Waist Circumference, HC: Hip Circumference, WHR: Waist-to-Hip Ratio, WHtR: Waist-to-Height Ratio, TC: Total Cholesterol, TG: Triglycerides, LDL-c: Low Density Lipoprotein cholesterol, HDL-c: High Density Lipoprotein cholesterol, EH: Eating Habits, EDU: Education. OCP: Occupation, AI: Average income, AlI: Alcohol intake, PIA: Physical inactivity.

### Diabetic population characteristics

In Table 5, A total of 1507 diabetic patients (513 rural participants and 994 urban participants) were enrolled with a media age of 53.7 years (IQR, 46.2–61.4). With respect to age distribution (p = 0.011), occupation (p < 0.001), level of education (p < 0.001), average income (p < 0.001), physical inactivity (p < 0.001), smoking (p = 0.003), alcohol intake (p < 0.001), and eating habits (p = 0.042), significant differences were observed between urban and rural participants. There were no significant differences regarding gender distribution (p = 0.239), marital status (p = 0.908), intensity of performed physical activity (p = 0.287) and reached recommended amount of fruits and vegetables/day (p = 0.179).

**Table 5:** Socio-demographic characteristics of the diabetes population in urban and rural areas, median (IQR) or n (%).

Variables	Total (n = 1507)	Rural (n = 513)	Urban (n = 994)	p-value
**Age (years)**				
**Median (IQR)**	53.7 (46.2–61.4)	54.0 (46.2–62.0)	53.3 (46.2–61.0)	0.906
20–29 yrs n (%)	25 (1.7)	16 (3.1)	9 (0.9)	
30–39 yrs n (%)	97 (6.4)	37 (7.2)	60 (6.0)	
40–49 yrs n (%)	412 (27.3)	128 (25.0)	284 (28.6)	
50–59 yrs n (%)	524 (34.8)	173 (33.7)	351 (35.3)	
60+ yrs n (%)	449 (29.8)	159 (31.0)	290 (29.2)	0.011
**Gender n (%)**				
Male	924 (61.3)	304 (59.3)	620 (62.4)	
Female	583 (38.7)	209 (40.7)	374 (37.6)	0.239
**Familly history of diabetes n (%)**				
No	1037 (68.6)	392 (76.4)	645 (64.9)	
Yes	470 (31.2)	121 (23.6)	349 (35.1)	< 0.001
**Marital status n (%)**				
Single	56 (3.7)	17 (3.3)	39 (3.9)	
Married	1396 (92.6)	476 (92.8)	920 (92.6)	
Divorced	18 (1.2)	7 (1.4)	11 (1.1)	
Widowed	37 (2.5)	13 (2.5)	24 (2.4)	0.908
**Occupation n (%)**				
Formal	679 (45.2)	136 (26.7)	543 (54.7)	
Informal	281 (18.7)	189 (37.1)	92 (9.3)	
Unemployed	541 (36.0)	184 (36.1)	357 (36.0)	< 0.001
**Level of education n (%)**				
Illiterate	67 (4.5)	46 (9.0)	21 (2.1)	
Basic	246 (16.4)	136 (26.7)	110 (11.1)	
Secondary education	641 (42.7)	237 (46.6)	404 (40.7)	
Tertiary	548 (36.5)	90 (17.7)	458 (46.1)	< 0.001
**Average income (RMB) n (%)**				
<2000	307 (20.4)	172 (33.8)	135 (13.6)	
2000~4000	951 (56.7)	290 (57.0)	561 (56.5)	
4000~6000	256 (17.0)	33 (6.5)	223 (22.5)	
6000~8000	65 (4.3)	11 (2.2)	54 (5.4)	
≥8000	23 (1.5)	3 (0.6)	20 (2.0)	< 0.001
**Smoking n (%)**				
No	1070 (71.1)	390 (76.0)	680 (68.6)	
Yes	434 (28.9)	123 (24.0)	311 (31.4)	0.003
**Alcohol intake n (%)**				
No	1183 (78.6)	437 (85.2)	746 (75.2)	
Yes	322 (21.4)	76 (14.8)	246 (24.8)	< 0.001
**Physical activity on regular basis**				
No	980 (65.0)	373 (72.7)	607 (61.1)	
Yes	527 (35.0)	140 (27.3)	387 (38.9)	< 0.001
**Intensity of performed physical activity**				
High	31 (5.9)	12 (8.6)	19 (4.9)	
Moderate	42 (8.0)	11 (7.9)	31 (8.0)	
Low	454 (86.1)	117 (83.6)	337 (87.1)	0.287
**Eating habits**				
3 main courses/day	1158 (76.8)	410 (79.9)	748 (75.3)	
Irregular habits	349 (23.2)	103 (20.1)	246 (24.7)	0.042
**Reached recommended amount of fruits and vegetables/day**				
No	489 (32.5)	155 (30.3)	334 (33.7)	
Yes	1014 (67.5)	357 (69.7)	657 (66.3)	0.179

Data is presented as median (IQR) or n (%); Chi-square or Fisher’s test. p < 0.05 was considered significant difference. n: number. Physical activity on regular basis: Only participants reporting regular physical activity included in this analysis, ≥ 5 times/week at moderate or high intensity, Reached recommended amount of fruits and vegetables/day: 500 grams of fruits and vegetables/day.

### Metabolic risk factors for T2DM

Anthropometric variables and serum lipid levels of diabetes population between urban and rural areas are compared in Table 6. Significant differences in weight (p < 0.001), BMI (p *=* 0.016) and HDL-c (p < 0.001) were observed between participants of urban and rural areas. There were no significant differences with respect to systolic (p *=* 0.264) and diastolic BP (p *=* 0.429), total cholesterol (p *=* 0.790), triglycerides (p *=* 0.565), and LDL-c (p *=* 0.106) [Table 6].

**Table 6:** Anthropometric variables and serum lipid levels comparison of the diabetes population, median (IQR) or n (%).

Variables	Total (n = 1507)	Rural (n = 513)	Urban (n = 994)	p-value
**SBP (mmHg)**	116.5 (123–138)	125 (115–140)	121 (116.88–135)	0.264
**DBP (mmHg)**	70 (80–85)	80 (70–85)	80 (70–80.62)	0.429
**Hypertention**	475(31.5)	171(33.3)	304(30.6)	0.276
**Weight (Kg)**	69 (78–86)	76 (66–85)	80 (70–87.00)	<0.001
**Height (m)**	1.63 (1.69–1.73)	1.68 (1.62–1.73)	1.69 (1.64–1.74)	0.029
**BMI (Kg/m**^**2**^**)**	24.77 (27.37–29.98)	26.99 (24.05–30.00)	27.53 (25.09–29.94)	0.016
**Obesity (BMI ≥ 28 Kg/m**^**2**^**)**	648(43.1)	200(39.0)	448(45.3)	0.020
**WC(cm)**	69 (78–86)	98 (89.25–105.75)	99 (90–105)	0.099
**HC (cm)**	95 (102–110)	102 (93–110)	103 (95–109)	0.815
**WHR**	90 (99–105)	0.95 (0.91–1.03)	0.96 (0.91–1.04)	0.052
**TC (mmol/l) median (IQR)**	3.76 (4.48–5.23)	4.51 (3.73–5.20)	4.47 (3.77–5.24)	0.790
**TG(mmol/l) median (IQR)**	1.24 (1.79–2.65)	1.81 (1.23–2.62)	1.79 (1.25–2.66)	0.565
**HDL-c (mmol/l) median (IQR)**	0.77 (0.92–1.11)	0.87 (0.72–1.06)	0.95 (0.79–1.15)	<0.001
**LDL-c (mmol/l) median (IQR)**	2.23 (2.77–3.38)	2.74 (2.12–3.32)	2.78 (2.29–3.41)	0.106

### Glucose control and complications of the diabetic population

At least one chronic complication was diagnosed in 74.0% participants. Most patients had poor glycemic control (HbA1c >8%, 52.4%). Participants in the rural area were significantly more likely to have better glycemic control (p *=* 0.019) and tend to have less diabetes-related complications but no significant difference (72.2% versus 74.9%, p = 0.263). There was statistically significant difference in number of diabetes medications between rural and urban participants (p *=* 0.012), so was with the use of diabetes medications including biguanide, DPP4 inhibitor and glinides (p *=* 0.002, 0.004 and 0.024 respectively). Compared to rural participants, urban participants tended to develop large artery atherosclerosis, retinopathy and neuropathy (p *=* 0.035, 0.032 and 0.025 respectively) [Table 7].

**Table 7:** Blood glucose and complications of the diabetes population in this study, median (IQR) or n (%).

Variables	Total (n = 1507)	Rural (n = 513)	Urban (n = 994)	p-value
**FBS (mmol/l)**	8.51 (6.94–10.90)	8.55 (6.92–11.20)	8.45 (6.96–10.78)	0.554
**FBS (> 7mmol/l)**	1106 (73.6)	376 (73.3)	730 (73.7)	0.853
**HbA1c (%)**	8.00 (6.80–9.30)	7.90 (6.83–9.30)	8.00 (6.80–9.30)	0.900
**HbA1c (> 7%)**	968 (69.6)	356 (71.5)	612 (68.6)	0.263
**HbA1c (> 8%)**	789 (52.4)	247 (48.1)	542 (54.5)	0.019
**HbA1c (> 9%)**	406 (26.9)	141 (27.5)	265 (26.7)	0.732
**Diabetes Medications**				
None	495 (35.5)	203 (40.8)	292 (32.6)	
1	353 (25.3)	122 (24.5)	231 (25.8)	
2	366 (26.2)	120 (24.1)	246 (27.4)	
≥ 3	181 (13.0)	53 (10.6)	128 (14.3)	0.012
Biguanide	617 (44.0)	192 (38.6)	425 (47.0)	0.002
Sulfonylurea	88 (6.3)	24 (4.8)	64 (7.1)	0.088
Thiazolidenedione	10 (0.7)	1 (0.2)	9 (1.0)	0.089
Alpha glucosidase inhibitor	148 (10.6)	48 (9.6)	100 (11.1)	0.384
DPP4 inhibitor	41 (2.9)	6 (1.2)	35 (3.9)	0.004
Insulin	630 (44.6)	220 (43.8)	410 (45.1)	0.643
Exenatide or Liraglutide	7 (0.5)	1 (0.2)	6 (0.7)	0.236
Glinides	134 (9.6)	36 (7.2)	98 (10.9)	0.024
**Diabetes-related complications n (%)**				
None	363 (26.0)	138 (27.8)	225 (25.1)	
Yes	1031 (74.0)	359 (72.2)	672 (74.9)	
1	404 (29.1)	148 (29.8)	256 (28.5)	
2	285 (20.4)	104 (20.9)	181 (20.2)	
≥3	342 (24.5)	107 (21.5)	235 (26.2)	0.263
**Macrovascular complications**				
CAD	469 (33.4)	156 (31.3)	313 (34.5)	0.221
Stroke/TIA	156 (11.2)	63 (12.7)	93 (10.4)	0.192
Large artery atherosclerosis	539 (38.6)	174 (34.9)	365 (40.7)	0.035
**Microvascular complications**				
Retinopathy	353 (25.2)	109 (21.9)	244 (27.1)	0.032
Nephropathy	98 (7.0)	31 (6.2)	67 (7.5)	0.384
Neuropathy	540 (38.7)	173 (34.7)	367 (40.8)	0.025
**DFD**	21 (1.5)	11 (2.2)	10 (1.1)	0.108

Data is presented as median (IQR) or n (%); Chi-square or Fisher’s test. p < 0.05 was considered significant difference. n: number, DFD: Diabetic Foot Disease.

### Metabolic risk factors for glucose control and complications

We compared selected metabolic risk factors by the presence of diabetic complications and settlements (rural or urban). In the rural diabetics, those with diabetic complications had higher prevalence of FBS (> 7mmol/l), HbA1c (> 9%), obesity, triglycerides (≥ 1.71mmol/l) and irregular eating habits compared to those with non-diabetics. In the urban diabetics, there were significant differences regarding metabolic risk factors including FBS (>7mmol/l), obesity, HbA1c (>9%) and irregular eating habits [Table 8].

**Table 8:** Characteristics of patients with diabetes, according to presence or absence of complications and area.

Variables	Rural diabetics				Urban diabetics			
	Diabetic complications				Diabetic complications			
	Total (n = 513)	No (n = 138)	Yes (n = 375)	p-value	Total (n = 994	No (n = 255)	Yes (n = 739)	p-value
**FBS (> 7mmol/l)**	365 (73.4)	90 (65.2)	275 (76.6)	0.01	664 (74.1)	151 (67.1)	513 (76.5)	0.006
**HbA1c(> 7%)**	355 (71.4)	106 (76.8)	249 (69.4)	0.1	612 (68.6)	142 (64.3)	470 (70.0)	0.108
**HbA1c(> 8%)**	232 (46.7)	58 (42.0)	249 (48.5)	0.198	445 (49.6)	104 (46.2)	341 (50.7)	0.24
**HbA1c(> 9%)**	141 (28.4)	23 (16.7)	249 (32.9)	< 0.001	265 (29.5)	48 (21.3)	217 (32.3)	0.002
**Overweight (BMI ≥ 25 Kg/m**^**2**^**)**	147 (48.5)	46 (46.5)	101 (49.5)	0.619	259 (53.8)	72 (53.3)	187 (54.0)	0.888
**Obesity (BMI ≥ 28 Kg/m**^**2**^**)**	194 (55.4)	39 (42.4)	155 (60.1)	0.003	412 (65.0)	86 (57.7)	326 (67.2)	0.034
**TC(≥ 5.20 mmol/l)**	124 (25.0)	30 (21.7)	94 (26.3)	0.298	238 (26.6)	56 (24.9)	182 (27.1)	0.511
**TG (≥ 1.71mmol/l)**	261 (52.6)	54 (39.1)	207 (57.8)	< 0.001	463 (51.7)	107 (47.6)	356 (53.1)	0.153
**HDL-c (< 1.04 mmol/l)**	362 (73.0)	95 (68.8)	267 (74.6)	0.197	578 (64.5)	139 (61.8)	439 (65.4)	0.322
**LDL-c (≥ 3.38 mmol/l)**	113 (22.8)	30 (21.7)	83 (23.2)	0.731	237 (26.5)	53 (23.6)	184 (27.4)	0.255
**Irregular eating habits**	102 (20.5)	20 (14.5)	82 (22.8)	0.039	239 (26.6)	47 (20.9)	192 (28.6)	0.024	

Data is presented as n(%); Chi-square or Fisher’s test. p < 0.05 was considered significant difference. BMI: Body Mass Index, TC: Total Cholesterol, TG: Triglycerides, HDL-c: High Density Lipoprotein cholesterol, LDL-c: Low Density Lipoprotein cholesterol.

After adjusting for gender and age, differences in some risk factors between rural and urban diabetic participants remained significant. Urban participants were more likely to have risk factors of diabetic complications including obesity, HDL-c (< 1.04 mmol/l), physical inactivity and irregular eating habits [Table 9].

**Table 9:** Multivariable association of diabetic complication control with selected risk factors (reference: rural population).

Risk factors	OR (95% CI)	p- value	Adj. OR-gender (95% CI)	p- value	Adj. OR-gender and age (95% CI)	p- value
**FBS (> 7mmol/l)**	1.148(0.883–1.491)	0.303	1.136(0.874–1.477)	0.341	1.139(0.876–1.482)	0.330
**HbA1c (> 7%)**	0.800(0.622–1.030)	0.083	0.801(0.622–1.031)	0.086	0.798(0.620–1.028)	0.080
**Overweight (BMI** ≥ **25 Kg/m**^**2**^**)**	1.187(0.878–1.605)	0.265	1.165(0.860–1.578)	0.325	1.160(0.855–1.572)	0.340
**Obesity (≥ 28 Kg/m**^**2**^**)**	1.384(1.034–1.852)	0.029	1.370(1.023–1.834)	0.035	1.369(1.022–1.833)	0.035
**WC (men ≥ 90 cm; women ≥ 85 cm)**	1.323(0.952–1.838)	0.096	1.316(0.947–1.831)	0.102	1.318(0.948–1.833)	0.101
**WHR (men > 0.90; women > 0.85)**	1.006(0.683–1.482)	0.976	1.039(0.702–1.537)	0.849	1.037(0.701–1.535)	0.854
**Total cholesterol (≥ 5.20 mmol/l)**	0.846(0.574–1.249)	0.400	0.853(0.578–1.259)	0.424	0.855(0.579–1.261)	0.429
**Triglycerides (≥ 1.71mmol/l)**	0.983(0.772–1.252)	0.890	0.984(0.772–1.253)	0.893	0.985(0.773–1.255)	0.903
**HDL-c (< 1.04 mmol/l)**	0.668(0.518–0.862)	0.002	0.648(0.499–0.840)	0.001	0.648(0.499–0.841)	0.001
**LDL-c (≥ 3.38 mmol/l)**	1.264(0.858–1.864)	0.236	1.266(0.858–1.867)	0.235	1.259(0.854–1.857)	0.245
**Physical inactivity**	0.615(0.481–0.788)	<0.001	0.616(0.481–0.788)	<0.001	0.619(0.483–0.793)	<0.001
**Irregular eating habits**	1.410(1.077–1.846)	0.012	1.400(1.069–1.834)	0.014	1.412(1.077–1.851)	0.013

OR: Odds ratio. Adj: adjusted. Compared using multivariate logistic regression. p < 0.05 was considered significant difference.

## Discussion

With the urbanization, the burden of non-communicable diseases such as T2DM is being increased. While the prevalence of diabetes rising across all over the world, a higher growing trend was observed in developing countries, especially in rural areas. The global rural prevalence of diabetes was 5.7% (3.5–7.9) during 1985–1989 and 8.7% (6.8–10.7) during 2005–2011 [13]. In 2009, the prevalence of diagnosed T2DM in rural adults in Shanghai, China increased from 6.1% in 2002–2003 to 9.8% in 2009 [20]. Prevalence of diabetes is driven primarily by accompanying metabolic risk factors such as smoking, physical inactivity, overweight or obesity, dyslipidaemia, uric acid and harmful alcohol intake [38–43].

In this study, differences between participants residing in urban and rural settlements as for metabolic risk factors were described, as well as glycemic control and complications in diabetic participants enrolled in both areas. It is however noteworthy that along with the observed differences in prevalence of type 2 diabetes prediabetes and metabolic risk factors including hypertension and hyperlipidemia, we recorded more male subjects in the rural community and more females in the urban community. Indices of overweight and obesity (BMI and WHR) were observed higher in urban residents, as previous studies had shown [11,44,45]. At the time of survey, urban participants were still heavily impacted by unhealthy lifestyles involving physical inactivity and alcohol intake excessively although they had higher literacy, more employment and income. The higher prevalence of being overweight and/or obesity in urban residents might be ascribed to unhealthy lifestyles including physical inactivity and harmful alcohol intake.

Compared to urban residents, rural residents had higher concentration of LDL-c, and lower concentration of HDL-c. The differences of dyslipidaemia were confirmed to be significant different metabolic risk factors between urban and rural areas including HDL-c (< 1.04 mmol/l) and triglycerides (≥1.71mmol/l). Previous studies on the difference of HDL-c between urban and rural residents remained controversial [35, 38, 40, 42–44]. In this study, significant difference of the HDL-c was confirmed. It is noteworthy that among all participants, amounting to 38.8% had low HDL cholesterol.

Overweight, obesity and physical inactivity are important risk factors for diverse chronic medical conditions, including hypertension, dyslipidaemia and diabetes. In this study [3,4,44], we confirmed positive correlations between anthropometric variables, and serum lipids and blood pressure in both areas studied. Diabetic participants of urban residents in this study appeared to be less healthy than those enrolled in rural residents. The results showed that the median HbA1c level among diabetics in both regions was high, with no difference between rural and urban areas. And 69.6% of these subjects had bad control of diabetes as defined as HbA1c level > 7%. In urban area, more than 54.5% of their diabetic patients had poorly controlled diabetes as defined as HbA1c level ≥ 8%, while so was 48.1% of their patients in rural area, and similar results were found in Wuxi city, China [46]. Compared to rural diabetic participants, the urban diabetic participants had a higher prevalence of smoking, alcohol intake, physical inactivity, dyslipidaemia and irregular eating habits. Other predictors of glycemic control in terms of gender, history of hypertension and intensity of performed physical activity, there were no statistically significant differences between rural and urban diabetic participants. It is noteworthy that urban residents had better socioeconomic status as indicators of education, income and occupation were significantly better than rural residents. Recent studies showed that knowledge and awareness about diabetes in rural areas were poor and these may contribute to poor glycemic control for rural residents [47]. And future work will focus on the level of awareness and knowledge of diabetes in the general population as well as diabetic subjects in Xinjiang, China.

The prevalence of T2DM of urban residents wase found, in most studies, significantly higher than that of rural residents [20,39,48], while little literacy focused on the prevalence of the total diabetic complications. In this study, 74.0% of the T2DM registered were suffering from at least one diabetic chronic complication. And out of expect, no significant difference regarding the prevalence of T2DM complications in urban participants and rural participants was detected (72.2% versus 74.9%, P = 0.263).

It was apparent that poor glycemic control (HbA1c>9%), obesity and dyslipidaemia were contributing factors for the diabetic complications as found in this study. There were significant differences with respect to FBS (>7mmol/l), HbA1c (>9%), HDL-c (< 1.04 mmol/l) and irregular eating habits amongst participants with no diabetic complications and those combined with diabetic complications in both urban and rural areas.

Xinjiang Uygur Autonomous Region is located in the northwest of China and composed of more than 13 ethnic groups. The population density of was 4.42‰, and Uygur account for 46%, Han account for 40%, and Kazak account for 7%. The rural population of Xinjiang amounts to12.04 million with per capita disposable income of 5442 RMB [28]. Most rural residents were of low socioeconomic status and acquired little health knowledge awareness, as indicated in this study, and need long distance to the nearest medical institutions [28]. And this may contribute to the prevalence of diabetic complications.

It was highlighted by previous studies [10,49] that obesity was a major risk factor for prevalence of T2DM and glycemic control, so the diabetic complications were expected to be higher prevalence among obese participants. With the expectation, we found a higher prevalence obesity in urban and rural diabetic participants with complications. And these findings confirm that obesity is a major/traditional contributor to complications of T2DM in urban and rural areas.

### Limitations

Several limitations deserve to be mentioned. First, cross-sectional nature of this study determines that all participants in both urban and rural areas are not followed up, thus we don’t know the impact of changes in lifestyle on the epidemic of diabetes and the related complications threatens. Second, current study may not necessarily reflect the true prevalence of T2DM and related complications at a province or national level, since participants were recruited only in the First Affiliated Hospital of Xinjiang Medical University. Third, information of participants were obtained from patient medical records or via participant self-report, thus, results might be adversely affected by recall bias or social desirability bias. Finally, uric aicd is considered as an important metabloic risk factor and Ivonne Sluijs et al., [43] showed that higher uric acid was associated with a higher diabetes risk. The prevalence of hyperuricemia in Uygur subjects in Xinjiang was 8.2% [50]. The confirmation of the correlation between serum uric acid level and a variety of metabolic parameter however was not designed in this study, although this was supported by our previous study [50].

## Conclusions

In summary, the present study provides a snapshot of the current situation of metabolic risk factors and complications of T2DM in both urban and rural communities in Xinjiang, China. There is an urgent need to avert the unhealthy lifestyle, and those people with high risk factors are recommended keeping healthy normal weight and developing a physically active lifestyle. There is an urgent need to increase awareness for approaches for the prevention and better management of T2DM and its complications with urbanization especially in developing countries.
